# Increased Angiogenesis by Exosomes Secreted by Adipose-Derived Stem Cells upon Lipopolysaccharide Stimulation

**DOI:** 10.3390/ijms22168877

**Published:** 2021-08-18

**Authors:** Shao-Chun Wu, Pao-Jen Kuo, Cheng-Shyuan Rau, Lien-Hung Huang, Chia-Wei Lin, Yi-Chan Wu, Chia-Jung Wu, Chia-Wen Tsai, Ting-Min Hsieh, Hang-Tsung Liu, Chun-Ying Huang, Ching-Hua Hsieh

**Affiliations:** 1 Department of Anesthesiology, Kaohsiung Chang Gung Memorial Hospital and Chang Gung University College of Medicine, Kahosiung 83301, Taiwan; shaochunwu@gmail.com; 2 Department of Plastic and Reconstructive Surgery, Kaohsiung Chang Gung Memorial Hospital and Chang Gung University College of Medicine, Kahosiung 83301, Taiwan; bow110470@gmail.com (P.-J.K.); sallylin1201@gmail.com (C.-W.L.); janewu0922@gmail.com (Y.-C.W.); alicewu8818@gmail.com (C.-J.W.); flying011401@gmail.com (C.-W.T.); 3 Department of Neurosurgery, Kaohsiung Chang Gung Memorial Hospital and Chang Gung University College of Medicine, Kahosiung 83301, Taiwan; ersh2127@adm.cgmh.org.tw (C.-S.R.); ahonbob@gmail.com (L.-H.H.); 4 Department of Trauma Surgery, Kaohsiung Chang Gung Memorial Hospital and Chang Gung University College of Medicine, Kahosiung 83301, Taiwan; hs168hs168@gmail.com (T.-M.H.); htl1688@cgmh.org.tw (H.-T.L.); 5 Center for Vascularized Composite Allotransplantation, Chang Gung Memorial Hospital, Taoyuan 33305, Taiwan

**Keywords:** angiogenesis, endothelial cell, exosome, adipose-derived stem cells, lipopolysaccharide, proteomic analysis, cAMP response element binding protein, nuclear factor-κB, activating protein 1, interleukin-8

## Abstract

Exosomes secreted by adipose-derived stem cells (ADSCs) enhance angiogenesis and wound healing. However, in clinical settings, wounds may be infected by various bacteria or pathogens. We investigated whether human ADSCs stimulated with lipopolysaccharide (LPS) secrete exosomes (ADSC-LPS-exo) that augment the angiogenesis of human umbilical vein endothelial cells (HUVECs). ExoQuick-TC exosome precipitation solution was used to purify exosomes from human ADSC culture media in the presence or absence of 1 µg/mL LPS treatment for 24 h. The uptake of ADSC-LPS-exo significantly induced the activation of cAMP response element binding protein (CREB), activating protein 1 (AP-1), and nuclear factor-κB (NF-κB) signaling pathways and increased the migration of and tube formation in HUVECs. RNA interference with CREB, AP-1, or NF-κB1 significantly reduced the migration of and tube formation in HUVECs treated with ADSC-LPS-exo. An experiment with an antibody array for 25 angiogenesis-related proteins revealed that only interleukin-8 expression was significantly upregulated in HUVECs treated with ADSC-LPS-exo. In addition, proteomic analysis revealed that eukaryotic translation initiation factor 4E, amyloid beta A4 protein, integrin beta-1, and ras-related C3 botulinum toxin substrate 1 may be potential candidates involved in ADSC-LPS-exo-mediated enhanced angiogenesis.

## 1. Introduction

Cell-based therapies, such as those comprising adipose-derived stem cells (ADSCs), are considered promising for improving wound healing [1], even in difficult situations such as chronic diabetic wounds [2,3] and irradiated wounds [4,5]. ADSCs have also been identified within subcutaneous tissues [6], because they play a pivotal role in maintaining the structure of skin tissues [7] and improving skin repair and regeneration [8,9]. In addition, ADSCs secrete a rich secretome to enhance cell differentiation, proliferation, migration, and tissue regeneration in the cellular microenvironment [10,11,12,13]. From the secretome, the exosomes secreted by ADSCs (ADSC-exo) are considered the main components of paracrine signaling and the main contributors to stem cell efficacy [14]. ADSC-exo has been demonstrated to accelerate cutaneous wound healing by promoting vascularization, tissue regeneration, proliferation, and the re-epithelialization of skin cells [13,15,16,17].

Exosomes are small lipid bilayer vesicles that are 305–150 nm in diameter and function to mediate intercellular communication by transporting proteins, nucleic acids, and lipids into the recipient cells, thus changing the behavior of the target cells [18,19]. Many clinical studies have demonstrated that exosomes secreted by autologous or allogeneic mesenchymal stem cells (MSCs) and ADSCs can enhance the healing process of chronic wounds by inducing angiogenesis and tissue regeneration [20,21]. Exosomes released by MSCs can promote bone regeneration by enhancing angiogenesis [22]. In addition, exosomes released from educated MSCs accelerate cutaneous wound healing by promoting angiogenesis [23,24]. However, in clinical settings, wounds may be infected by various bacteria. Even though the ADSC-exo-loaded alginate hydrogel [25] or engineered ADSC-exo [15,26] have been used to promote wound healing, one main question remains unanswered and less explored: whether the exosomes secreted by ADSCs in the absence or presence of various stimulators inside the infected wound present the same ability to enhance angiogenesis and promote wound healing. One of the most studied bacterial surface molecules is the glycolipid, lipopolysaccharide (LPS), which is produced by most Gram-negative bacteria [27,28]. Many Gram-negative bacterial species, such as *E. coli* [29], *P. aeruginosa* [30,31] and *S. marcescens* [30], which secrete LPS, commonly infect chronic wounds. LPS is a bacterial endotoxin component responsible for wound infection; therefore, the present in vitro study was designed to determine whether exosomes secreted by ADSCs following LPS stimulation (ADSC-LPS-exo) could enhance the angiogenesis of endothelial cells compared with ADSC-exo. In addition, we employed isobaric tags for the relative and absolute quantitation (iTRAQ) of the protein content of the designed exosomes to determine the potential exosomal proteins mediating the effect and mechanism of action.

## 2. Results

### 2.1. Characterization of Isolated Exosomes

**Figure 1:**
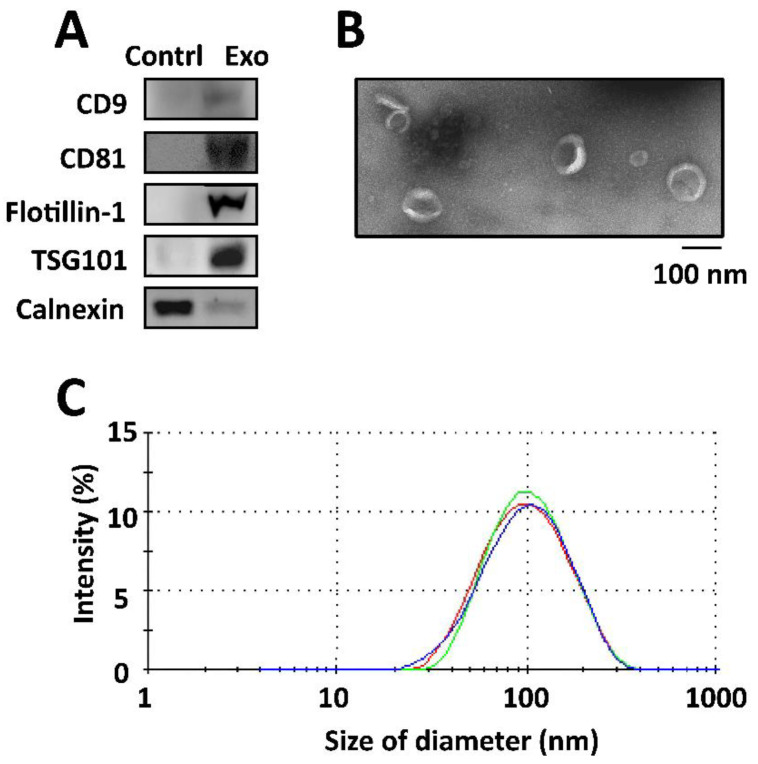
Characterization of exosomes isolated by (**A**) Western blotting for determining the exosomal surface markers of those proteins isolated from the culture medium and exosomes secreted by ADSCs. The selected positive exosomal surface markers included CD9, CD81, flotillin-1, and TSG101. Expression of calnexin was detected as negative control protein for the isolation of exosomes. (**B**) Transmission electron microscopy image displaying a cup-shaped appearance of the exosomes with lipid bilayers, and (**C**) the measurement of particle diameter and size distribution by dynamic light scattering (DLS) in triplicates.

When compared with proteins isolated from the culture medium, the isolated exosomes secreted by ADSCs expressed positive exosomal surface markers, including CD9, CD81, flotillin-1, and TSG101, with no expression of the negative control protein calnexin, as determined by Western blotting (Figure 1A). Transition electron microscopy (TEM) revealed that the exosomes displayed a cup-shaped appearance and were composed of lipid bilayers with acceptable quality in terms of size range and morphology (Figure 1B). An average size of 96.8 ± 39.7 nm was found in the measurements of size distribution of the exosomes in dynamic light scattering (DLS), with a single peak distribution and a PDI of approximately 0.76 (Figure 1C). The quality of the isolated exosomes was good, with a relatively uniform size distribution.

### 2.2. Uptake of ADSC-exo into HUVECs

**Figure 2:**
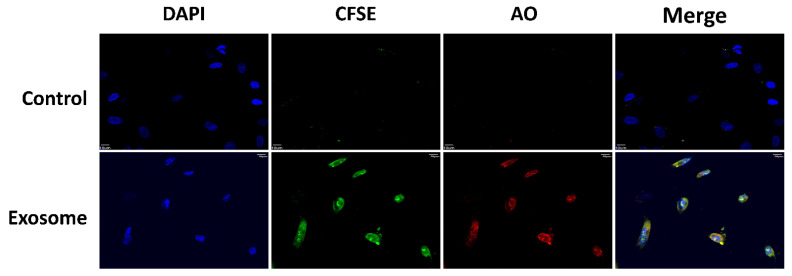
Confocal imaging of the uptake of labeled, purified ADSC-exo (30 µg) by HUVECs (Exosome) against those cells treated with medium (Control). The green fluorescence of CFSE and red fluorescence of AO found inside the cells indicated the uptake of protein and RNAs, respectively, by the ADSC-exo into the HUVECs. DAPI: 4′,6-diamidino-2-phenylindole; CFSE: carboxyfluorescein succinimidyl diacetate ester; AO: acridine orange.

The uptakes of labeled exosomes by the cultured HUVECs were found following the incubation of the exosomes with HUVECs for 24 h (Figure 2). The green fluorescence of CFSE and red fluorescence of AO found inside the cells revealed the uptake of protein and RNAs, respectively, of the ADSC-exo into the HUVECs.

### 2.3. Induction of Signal Transduction Pathways after ADSC-LPS-exo Treatment

**Figure 3:**
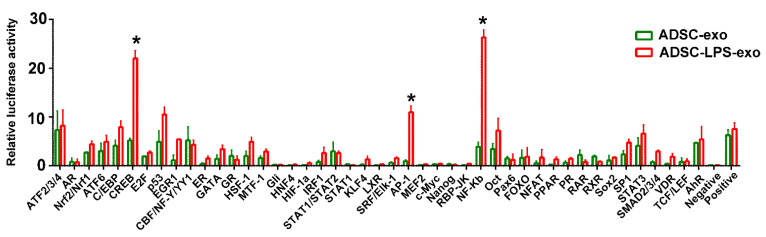
Activated signal transduction pathways in Cignal Finder Reporter Arrays at 24 h after the uptake of ADSC-LPS-exo into HUVECs, compared to those treated with ADSC-exo. The reporter array had 45 inducible transcription factor-responsive constructs, which controlled firefly luciferase reporter gene expression, in duplicate, in a 96-well plate. The *Renilla* luciferase gene was included in the wells for the normalization of transfection efficiency. (*, a *p*-value < 0.05 with at least 5-fold expression of the luminescence).

**Figure 4:**
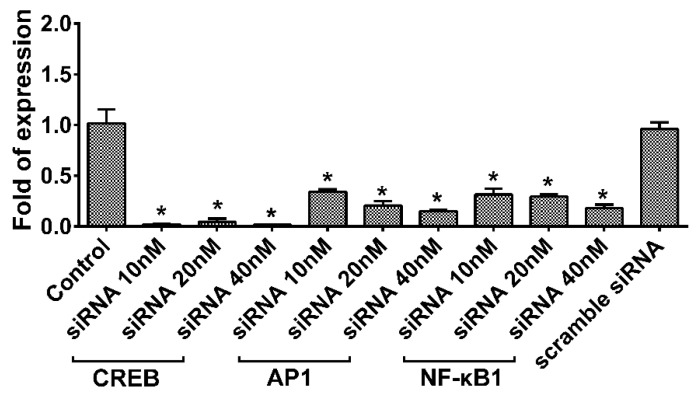
RT-qPCR was performed to validate the effective knockdown of the CREB, AP-1 and NF-κB1 genes of HUVECs by the transfection of pooled siRNAs for CREB, AP-1, and NF-κB1 from 10 nM to 40 nM, followed by 30 µg ADSC-LPS-exo treatment for 24 h. The HUVECS treated with ADSC-LPS-exo alone were used as controls. The HUVECs treated with scramble siRNA first, followed by ADSC-LPS-exo treatment were used as mock control. *n* = 6 for each condition. (*, a *p*-value < 0.05 with at least 2-fold reduced expression of the gene).

As shown in Figure 3, the experiments with Cignal Finder Reporter Arrays in HUVECs, which could detect 45 signal transduction pathways encoded by firefly luciferase reporter genes, revealed that the uptake of ADSC-LPS-exo significantly induced the activation of CREB, AP-1, and NF-κB signal transduction pathways in cells transfected with ADSC-exo. The expression of CREB, AP-1, and NF-κB1 genes in HUVECs following ADSC-LPS-exo treatment was effectively knocked down by the transfection of pooled siRNAs for CREB, AP-1, and NF-κB1 (Figure 4).

### 2.4. Angiogenesis after ADSC-LPS-exo Treatment

**Figure 5:**
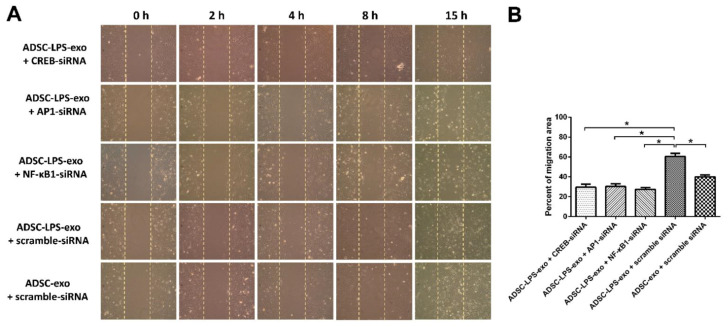
(**A**), Cell migration into the gap created by Culture-Inserts in the culture plate coated with 1% gelatin for HUVECs transfected with 10 nM siRNA-CREB, siR-NA-AP-1, siRNA-NF-κB1, or scramble siRNAs. The silicone insert was carefully removed 24 h later, leaving a 500 μm cell-free gap. Thereafter, 30 μg ADSC-exo or ADSC-LPS-exo was added to the wells. An Olympus CKX41 microscope (Olympus) was used to capture the migration distance of cells to the cell-free zone at 0, 2, 4, 8, and 15 h. Image J was used to calculate the area of migration of cells. (**B**), The bar plot indicates the percentage of migration area of the gap measured at 8 h following exosome treatment. *n* = 6 for each condition. (*, a *p*-value < 0.05 with at least a 2-fold difference).

**Figure 6:**
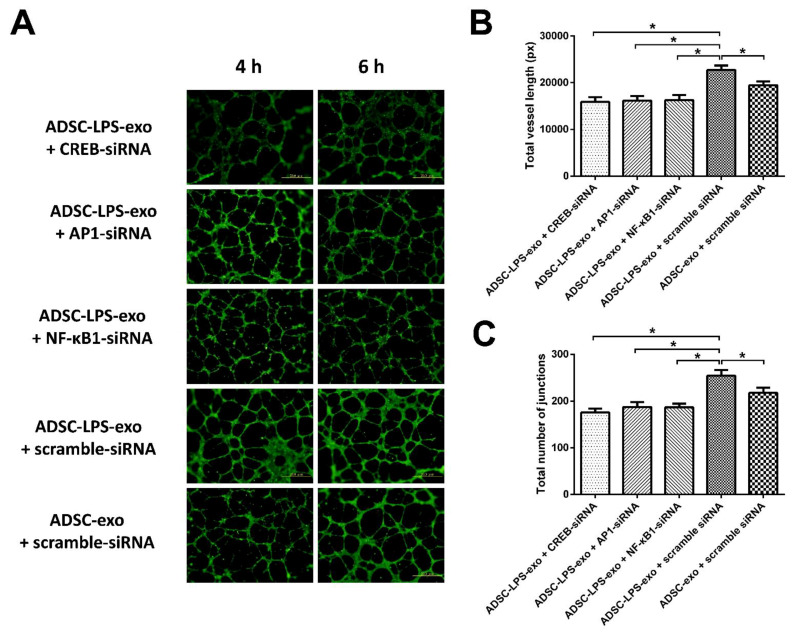
(**A**), After the seeding of FBS-MEM199 at a density of 1 × 10^4^ cells/well in 100 μL culture medium onto a 96-well plate coated with 50 μL Matrigel per well, tube formation was detected in the HUVECs transfected with 10 nM siRNA-CREB, siR-NA-AP-1, siRNA-NF-κB1, or scramble siRNAs, followed by ADSC-LPS-exo (30 µg) treatment as well as in those HUVECs transfected with scramble siRNA and treated with ADSC-exo (30 µg). (**B**), The bar plots indicate the total vessel length measured by Angiotool at 6 h following exosome treatment. (**C**), The bar plots indicate the total number of junctions measured by Angiotool at 6 h following exosome treatment. *n* = 6 for each condition. (*, a *p*-value < 0.05 with at least a 2-fold difference).

In comparison with the HUVECs transfected with scramble siRNAs and treated with ADSC-exo, the cell migration and tube formation of HUVECs transfected with 10 nM siRNA-CREB, siRNA-AP-1, siRNA-NF-κB1, or scramble siRNAs, followed by ADSC-LPS-exo treatment, were measured. The results revealed that HUVECs treated with ADSC-LPS-exo had significantly enhanced cell migration (Figure 5) and tube formation (Figure 6), which manifested as increased total vessel lengths and the total number of junctions detected by Angiotool, compared to those treated with ADSC-exo. In addition, transfection with siRNA-CREB, siRNA-AP-1, and siRNA-NF-κB1 significantly decreased the enhanced cell migration and tube formation following ADSC-LPS-exo treatment.

### 2.5. Expression of Angiogenesis-Related Proteins

**Figure 7:**
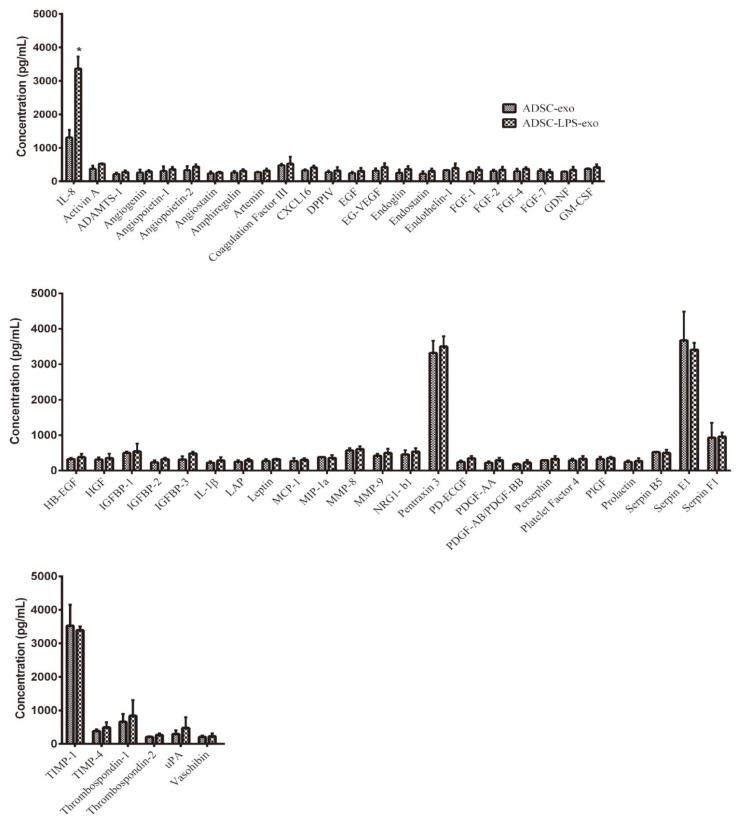
Relative expression of angiogenesis-related proteins in the cell lysates of HUVECs following treatment with 30 µg ADSC-LPS-exo or ADSC-exo was detected in quadruplicates using The Proteome Profiler Human Angiogenesis Antibody Array, which can detect 25 human angiogenesis-related proteins simultaneously with chemiluminescent detection reagents (*, a *p*-value < 0.05 with at least a 2-fold difference).

The Proteome Profiler Human Angiogenesis Antibody Array was used to measure the expression of angiogenesis-related proteins in HUVECs following treatment with 30 µg ADSC-LPS-exo against those treated with ADSC-exo at the same dosage. Of the measured 25 human angiogenesis-related proteins, only interleukin-8 (IL-8) was significantly upregulated (Figure 7), by around threefold in HUVECs following treatment with ADSC-LPS-exo compared to ADSC-exo. There were no significant changes in the other 52 angiogenesis-related proteins between HUVECs treated with ADSC-LPS-exo and ADSC-exo.

**Figure 8:**
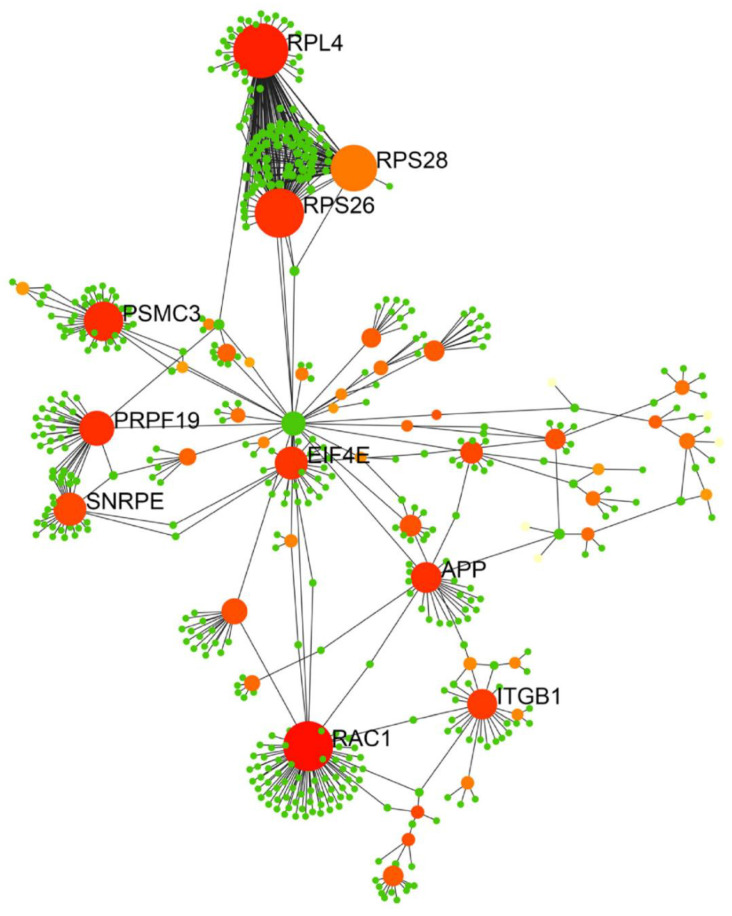
PPI network of 168 exosomal proteins (88 upregulated and 80 downregulated in ADSC-LPS-exo vs. ADSC-exo) identified from the iTRAQ experiments. The PPI networks were constructed by Cytoscape presenting the hub proteins, known to possess the highest degree of connectivity among the protein targets. PPI: protein–protein interaction.

An iTRAQ-based quantitative proteomic analysis was used to analyze the expression of exosomal proteins in the ADSC-exo and ADSC-LPS-exo samples (*n* = 2). The exosomes were labeled with 4-plex iTRAQ reagents of varying masses (114–117). In total, 1190 proteins were identified, with 168 exosomal proteins having more than 2-fold dysregulated expression (Supplementary File S1). Of these 168 exosomal proteins, 88 were upregulated and 80 were downregulated in ADSC-LPS-exo vs. ADSC-exo. The biological functions of the upregulated genes were determined using the Gene Ontology and Kyoto Encyclopedia of Genes and Genomes databases. The upregulated genes were particularly enriched in the top ten pathways (Supplementary File S2): metabolic pathways (23 proteins), PI3K-Akt signaling pathway (14 proteins), ECM–receptor interaction (12 proteins), focal adhesion (11 proteins), human papillomavirus infection (10 proteins), pathways in cancer (10 proteins), proteoglycans in cancer (9 proteins), protein digestion and absorption (8 proteins), carbon metabolism (8 proteins), and phagosome (7 proteins). We imported the PPI data into Cytoscape and constructed a PPI network of exosomal proteins (Figure 8) to identify the top ten hub proteins, defined as proteins with the highest degree of connectivity. These ten hub proteins included 60S ribosomal protein L4 (RPL4), 40S ribosomal protein S28 (RPS28), 40S ribosomal protein S26 (RPS26), 26S protease regulatory subunit 6A (PSMC3), pre-mRNA-processing factor 19 (PRPF19), small nuclear ribonucleoprotein E (SNRPE), eukaryotic translation initiation factor 4E (EIF4E), amyloid beta A4 protein (APP), integrin beta-1 (ITGB1), and ras-related C3 botulinum toxin substrate 1 (RAC1). Among these, four proteins, EIF4E, APP, ITGB1, and RAC1, are known to be involved in angiogenesis.

## 3. Discussion

Many studies have demonstrated that ADSCs can enhance wound healing in chronic wounds [32,33,34] or diabetic wounds [2,35]. Our findings revealed that pretreatment of ADSCs with LPS can produce exosomes carrying potent molecules for enhanced angiogenesis. This phenomenon is related to the activation of CREB, AP-1, and NF-κB signal transduction pathways and IL-8 production in recipient HUVECs during exosome treatment. Proteomic analysis of ADSC-LPS-exo revealed the notable presence of EIF4E, APP, ITGB1, and RAC1, which may be potential candidates involved in exosome-mediated enhanced angiogenesis.

NF-κB, AP-1, and CREB are known to play a pivotal role in angiogenesis because multiple transcription factor-binding sites for NF-κB, AP-1, CREB, and hypoxia-inducible factor (HIF) have been identified within the vascular endothelial growth factor (VEGF) promoter [36]. Although NF-κB and AP-1 are at the receiving end of different signaling pathways, they are often activated by the same stimuli and simultaneously regulate common target genes implicated in angiogenesis [37,38,39]. For example, NF-κB can mediate VEGF regulation via the AP-1 subunit c-Fos [40] and another AP-1 sub-unit, JunB [37]. The cAMP pathway is known to stabilize endothelial barrier function and maintain vascular physiology. CREB activation, with CREB binding to the VEGF promoter region, is essential for VEGF expression [41,42,43,44,45]. Promoter analysis revealed that the deletion of the CREB site in the proximal region of the promoter markedly reduced VEGF-induced promoter activity, whereas deletion of the upstream NF-κB site had a moderate effect [46]. In addition to angiogenesis [47], CREB is involved in multiple signaling pathways that regulate cell differentiation, proliferation, and migration [48].

IL-8 is a pro-inflammatory chemokine that belongs to the CXC sub-family and has been shown to enhance angiogenesis, increase proliferation and survival, and promote the migration of endothelial cells [49,50,51]. In addition, it correlates with angiogenesis in in vivo models [52,53]. The human IL-8 gene is transcriptionally regulated by NF-κB, AP-1, CREB, CAAT/enhancer-binding protein β (C/EBPβ, also known as NF-IL-6), and C/EBP homologous protein (CHOP) [54]. IL-8 expression is primarily regulated by NF-κB-and/or AP-1-mediated transcriptional activity [52,55]. In *Trichomonas vaginalis* infection, NF-κB and CREB are involved in IL-8 production in human neutrophils [56]. Human neutrophils may promote angiogenesis via a paracrine feedforward mechanism involving endothelial IL-8 [51]. The biological effects of IL-8 are mediated through the binding of IL-8 to two cell-surface G-protein-coupled receptors, termed as CXCR1 and CXCR2 [49,57,58], with a high affinity [59]. It has been reported that HUVECs constitutively express CXCR1 and CXCR2 mRNA and proteins. Recombinant human IL-8 induced endothelial cell proliferation and capillary tube formation, whereas the neutralization of IL-8 by anti-IL-8 antibody blocked IL-8-mediated capillary tube formation [50]. The mechanism of IL-8 angiogenesis regulation may be due to the enhanced proliferation and survival of endothelial cells by the differential expression of anti-apoptotic genes and, in part, by the activation of MMP-2 and MMP-9 [50].

Proteomic analysis revealed that EIF4E, APP, ITGB1, and RAC1 may be potential candidates for ADSC-LPS-exo-mediated enhanced angiogenesis. In eukaryotes, most mRNAs are translated in a cap-dependent manner. The mRNA 5′ cap-binding protein EIF4E is a key player in controlling mRNA translation, a critical process in regulating cell growth, proliferation, and differentiation. Overexpression of EIF4E results in a dramatic increase in VEGF and fibroblast growth factor-2 (FGF-2), two potent angiogenic agents [60,61]. In contrast, blocking EIF4E signaling selectively inhibited angiogenesis in human endothelial cells in vitro [62] and in vivo [63,64].

APP has been reported to be highly expressed in the endothelium of neoforming vessels [65]. The proteolytic cleavage product of APP by β-and γ-secretases mediates sprouting angiogenesis and the formation of new blood vessels [66]. In addition, inhibitors of β-and γ-secretases would inhibit angiogenesis [65].

ITGB1 is a membrane-anchored subunit of many integrins that serve as mechanosensory proteins in endothelial cells [67,68]. Integrins are heterodimeric transmembrane receptors that mediate cell–cell interactions and crosstalk between cells and the extracellular matrix [69]. ITGB1 is essential for blood vessel formation during embryonic development, postnatal vascular remodeling, and vessel maturation [70,71,72]. Loss- and gain-of-function studies have shown that endothelial ITGB1 is involved in angiogenesis and vessel wall remodeling [70,71]. Antibody blockades demonstrate that ITGB1 is functionally important for the migration of endothelial cells [73].

RAC1 is a GTPase that belongs to the RAS superfamily of small GTP-binding proteins (Rho GTPases), which act as molecular switches that transduce extrinsic stimuli into cytoskeletal rearrangements [74]. In endothelial cells, RAC1 controls cell migration and cell–cell junctions, thereby regulating the permeability and formation of vessels [75]. A study revealed that IL-8-upregulated RAC1 increased the migration of HUVECs [76]. In the early stage of metastasis, oncogenic cells undergoing epithelial–mesenchymal transition can communicate with endothelial cells via exosomal Rac1/PAK2 as angiogenic promoters [77].

Although recent reports have implicated exosomes to act in intercellular signaling, their effect in modulating signaling pathways in recipient cells is far from being completely elucidated. Outlining these complex networks may expand our knowledge of the underlying mechanisms involved in the function of exosomes under different stimuli for intercellular communication. However, the current understanding of these “discarded cargoes” is quite limited. EV cargo composition is complex and consists of hundreds to thousands of different proteins, unique lipids, some DNA and mRNA, microRNA, small nucleolar RNA, mitochondrial RNA, and long non-coding RNA (lncRNA) [78]. Although it has been implied that exosomal cargo appears to act in a combinatorial manner when communicating with the recipient cells [79], the molecular cargo mediates specific functions of the exosomes and they do so singly or in combination with other exosomal cargoes, which are still unknown. In addition, whether all exosome cargoes are selectively sorted upon various stimuli to act in different environments remains to be determined.

Furthermore, other limitations of this study should be acknowledged. First, 168 abundant exosomal proteins with 10 hub proteins were identified in ADSC-LPS-exo. The four proteins that are suggested to explain the function of angiogenesis were based on a literature search. It cannot be excluded that angiogenesis may be induced partly or synergistically by other exosomal proteins. In addition, Western blot analyses of each protein or phosphorylated protein with investigation of the function of these proteins on endothelial cells is necessary to confirm the conclusions. Moreover, the effect on angiogenesis by exosomes secreted from ADSCs upon LPS stimulation may rely on factors other than the protein cargo inside exosomes, such as microRNAs [80,81] or lncRNAs [82], which are known to mediate the function of ADSCs on angiogenesis, and should be considered accordingly. Furthermore, LPS, the stimulator used in this study, is only one of a common bacterial toxin component in infectious wounds. This study only investigated one endotoxin found in a complex bacterial wound. Thus, it is considerably different from the milieu that mimics a bacterial wound. There are many other bacterial or fungal components, such as lipoproteins, lipoteichoic acid (LTA; Gram-positive bacteria only), lipoarabinomannan (mycobacteria only), zymosan (yeast), single- or double-stranded RNAs, and flagellin, whose modulatory effects on exosomes secreted by ADSCs remain unexplored. As such, in vivo studies may be necessary to provide valuable information regarding the function of ADSC-exo in complex wounds.

## 4. Materials and Methods

### 4.1. Cultured Human ADSCs and Human Umbilical Vein Endothelial Cells (HUVECs)

Human ADSCs were purchased from Lonza (cat. #PT-5006, LOT No. 0000543947; Walkersville, MD, USA). These cells were expanded for subsequent passages using keratinocyte-SFM (17005-042, GIBCO-Invitrogen, Amarillo, TX, USA), and were supplemented with 2 mM N-acetyl-L-cysteine (A8199, Sigma-Aldrich, St. Louis, MI, USA), L-ascorbic acid 2-phosphate (A8960, Sigma-Aldrich), bovine pituitary extract, human recombinant EGF, and 5% fetal bovine serum (FBS; 16000044, GIBCO-Invitrogen). Prior to the experiments, the cells tested positive for stem cell markers CD29, CD36, CD73, CD44, CD90, and CD105, and negative for CD14, CD31, CD34, and CD45, by flow cytometry analysis.

HUVECs were purchased from the Bioresource Collection and Research Center (No. H-UV001, BCRC, Hsinchu, Taiwan) and cultured in medium 199 supplemented with 10% FBS, 25 U/mL heparin (H-3149, Sigma-Aldrich), 30 µg/mL endothelial cell growth supplement (ECGS, 02-102, Millipore, Billerica, MA, USA), 2 mM L-glutamine, 1.5 g/L sodium bicarbonate, and 1X penicillin/streptomycin. HUVECs between passages 4 and 8 were used in all the experiments. All cells were incubated at 37 °C in a humidified atmosphere containing 5% CO_2_.

### 4.2. Exosome Isolation

The exosomes were purified from the ADSC culture media in the presence or absence of 1 µg/mL LPS treatment for 24 h using ExoQuick-TC^TM^ exosome precipitation solution (EXOTC50A-1, System Biosciences, Palo Alto, CA, USA). The media were centrifuged at 3000× *g* for 15 min, and the supernatant was transferred into a new tube, followed by the addition of equal volumes of the ExoQuick-TC^TM^ solution. After mixing, supernatants were refrigerated at 4 °C overnight for at least 12 h and then centrifuged at 1500× *g* for 30 min. The supernatant was discarded, and the pellet was resuspended in PBS and used for further experiments.

### 4.3. Characterization of Exosomes

Under the guidelines of the Minimal Information for Studies of Extracellular Vesicles (MISEV2018) [83], characterization of isolated exosomes was performed to validate the expression of positive and negative exosomal surface markers, detect the morphology and bi-lipid layer structure, and measure the diameters and size distribution of the exosomes. With the culture medium used as a control, Western blotting for the expression of exosomal surface markers of the isolated exosomes was performed in triplicates. The total protein of exosomes was separated by polyacrylamide gel electrophoresis and electrotransferred to polyvinylidene fluoride (PVDF) membranes (Millipore, Billerica, MA, USA). To block the membrane, 5% skim milk in PBS/Tween-20 membranes was used, followed by the subsequent incubation of primary antibodies against four positive proteins for the exosomes, including CD9 (cat. #ab92726, 1:1000; Abcam, Cambridge, MA, USA), CD81 (cat. #ab109201, 1:1000; Abcam), flotillin-1 (cat. #18634, 1:1000; Cell Signaling Technology, Danvers, MA, USA), TSG101 (cat. #ab30871, 1:1000; Abcam). The primary antibody against calnexin (cat. #ab22595; 1:1000; Abcam) was used as a negative control protein, at 4 °C. Membranes were then washed with 0.1% TBS/Tween 20 for 10 min, three times. The incubation was performed at 37 °C with horseradish peroxidase (HRP)-conjugated secondary antibodies (cat. #NA931; GE Healthcare Amersham, Piscataway, NJ, USA) for 2 h. A FluorChem SP imaging system (Alpha Innotech, San Leandro, CA, USA) was used to detect and quantify the protein expression.

For the TEM analyses, we fixed 10 µL exosomes with 2.5% glutaraldehyde for 2 h and then added these exosomes to a 200 mesh Formvar with carbon stabilization. A transmission electron microscope HT-7700 (Hitachi, Tokyo, Japan) at 100 kV was used to analyze the exosome samples which were stained on grids with 2% uranyl acetate for 1 h.

The particle diameters of the isolated exosomes were measured using a Zetasizer Nano-ZS DLS system (Malvern, Montréal, QC, Canada) in triplicates. Briefly, each sample was loaded into an ultraviolet microcuvette (BRAND; Essex, CT, USA) in amounts of 100 µL at 4 °C. According to the fluctuations of the scattered light intensity detected at a wavelength of 633 nm and a fixed angle of 173°, the Brownian motion of each particle was measured. The peak of the Gaussian model fit to the particle distribution determined the average diameter of these exosomes and was presented by the polydispersity index (PDI) [84]. Each data point represented an average of three automatic measurements of 12–18 runs.

### 4.4. Exosome Uptake Using the Exo-Glow Kit

The uptake of exosomes by the cultured HUVECs was analyzed by labeling 30 µg of purified ADSC-exo using the Exo-Glow Kit (SBI Systems Bioscience, Palo Alto, CA, USA), according to the manufacturer’s instructions. The Exo-Glow Kit contained exo-Red (cat. #EXOR100A-1), which is based on membrane-permeable acridine orange (AO) chemistry using fluorescently labeled single-stranded RNAs inside exosomes (typical RFP filter set), and Exo-Green (cat. #EXOG200A-1), which is based on membrane-permeable carboxyfluorescein succinimidyl diacetate ester (CFSE); activated from inside the exosomes, it is coupled to the amino ends of proteins to fluoresce green (typical GFP filter set). The uptake of exosomes was measured by incubating the labeled exosomes with 1× 10^5^ cells per well in a 6-well culture plate in serum-free media for 30 min in a cell culture incubator. Subsequently, the cells were stained with 4′,6-diamidino-2-phenylindole (DAPI mounting medium VECTOR-H1200) and detected using a confocal microscope (FLUOVIEW FV10i, Olympus).

### 4.5. Involved Signal Pathways Following Exosome Delivery

The effects of ADSC-LPS-exo on 45 signal transduction pathways in HUVECs were elucidated using the Cignal Finder Reporter Arrays (cat. #CCA-901 L; Qiagen). The reporter array had 45 inducible transcription factor-responsive constructs, controlling the firefly luciferase reporter gene, in duplicates, in a 96-well plate. The *Renilla* luciferase gene was included in the wells to normalize the transfection efficiency. To determine the effects of exosomes on the signaling pathways, the constructs were reverse-transfected into HUVECs at 8 × 10^4^ cells per well in Opti-MEM^®^ (Invitrogen, cat. #31985-062) containing 10% FBS and 1% NEAA (Invitrogen, cat. #11140-050) using the Effectene Transfection Reagent (Qiagen, cat. #301425).

At 24 h post-transfection of HUVECs with ADSC-LPS-exo, the Dual-Glo^®^ Luciferase Assay System (Promega, cat. #E2940) was used to detect luciferase, according to the manufacturer’s instructions. A Hidex Sense microplate reader (Hidex, Turku, Finland) was used to measure luminescence. Luminescence measurements were compared between those treated with ADSC-LPS-exo and those treated with ADSC-exo. A negative control and positive control for luminescence were provided with the arrays. The pathway was considered significantly activated when there was a 5-fold expression of luminescence with a *p*-value < 0.05.

### 4.6. Inhibition of cAMP Response Element Binding Protein (CREB), Activating Protein 1 (AP-1), and Nuclear Factor-κB (NF-κB)

For RNA interference studies, a silencer siRNA duplex targeting the mRNA sequences of human CREB1 (AUUCUGUAGUUGCUUUCAG, AGACGGACCUCUCUCUUUC, UGAUUUGUGGCAGUAAAGG, and UUUAGCUCCUCAAUCAAUG), AP-1 (siRNA-AP-1, GUUCCUUCGUGCCCACGGU, GAAGGAACGUCUGGAGUUU, CAGUGACCAGCCUUCCGAU and CAGUCCUGUGUGAGGAUUA), and *NF-κB1* (siRNA-*NF-κB1*, GGGUAUAGCUUCCCACACU, CAGAGUUUACAUCUGAUGA, CAAUUUCCCACACCGUGUA and CUUAUGGUGGGAUUACUUU), and control siRNA duplexes (siRNA-scramble, GAUCAUACGUGCGAUCAGA), were purchased from Sigma-Aldrich. For siRNA transfection, 1 × 10^6^ cells were seeded into 10 cm dishes with complete medium at ∼70% confluence the day before transfection. Effectene transfection reagent (Qiagen, cat. #301425) was diluted with Opti-MEM at a ratio of 1:50. Sequentially diluted siRNA at concentrations of 10, 20, and 40 nm were incubated with the HUVECs for 20 min at room temperature to allow siRNA-Effectene transfection reagent complexes to form. Six hundred microliters of the complex was then added to wells containing HUVECs in Opti-MEM^®^ medium. The HUVECs were incubated at 37 °C in a 5% CO_2_ incubator for 6 h. The media containing the complexes were then removed and replaced with complete medium in the presence of 30 µg ADSC-exo or ADSC-LPS-exo for 24 h. The mRNA expression of CREB, AP-1, and NF-κB1 was assessed 24 h later. PCR primers were designed using the following sequences: CREB F, 5′-AAGCTGAAAGTCAACAAATGACAGTT-3′ and R, 5′-TGGACTGTCTGCCCATTGG-3′; AP-1 F, 5′-GTGAGAGATTTGCCAGGGTC-3′ and R, 5′-AGAGAGAAGCCGTCAGGTTG-3′; NF-κB1 F, 5′-ACACCGTGTAAACCAAAGCC-3′ and R, 5′-AGCCAGTGTTGTGATTGCT-3′; and GAPDH F, 5′-ACAGTCAGCCGCATCTTCTT-3′ and R, 5′-GCCCAATACGACCAAATCC-3′. This study was performed with *n* = 6 for each condition.

### 4.7. Cell Migration Assay

The migration assay was performed using two-well IBIDI^TM^ Culture-Inserts (Ibidi, Martinsried, Germany). The silicone culture inserts were then placed in the middle of a 12-well plate. The culture plate was coated with 1% gelatin, and HUVECs transfected with 10 nM siRNA-CREB, siRNA-AP-1, siRNA-NF-κB1, or scramble siRNAs were seeded onto the culture plate at a density of 1 × 10^4^ cells/mL in 70 μL volumes and allowed to proliferate to 100% confluence. The silicone insert was carefully removed 24 h later, leaving a 500 μm cell-free gap. Thereafter, ADSC-exo or ADSC-LPS-exo were added to the wells. An Olympus CKX41 microscope (Olympus) was used to capture the migration distance of cells to the cell-free zone at 0, 2, 4, 8, and 15 h. Image J was used to calculate the area of migration of cells. This study was performed with *n* = 6 for each condition.

### 4.8. Tube Formation Assay

HUVECs transfected with 10 nM siRNA-CREB, siRNA-AP-1, siRNA, NF-κB1, or scramble siRNAs in 2.5% FBS-MEM199 were seeded at a density of 1 × 10^4^ cells/well in 100 μL culture medium in a 96-well plate coated with 50 μL Matrigel per well and were incubated at 37 °C in an atmosphere containing 5% CO_2_. After treatment with 30 µg ADSC-exo or ADSC-LPS-exo per well, the tube formation of HUVECs at 4 and 6 h was captured in four independent experiments using an inverted phase contrast microscope (Olympus, IX71, Tokyo, Japan) at 10× magnification and quantified by Angiotool software [85] to calculate total vessel lengths and the total number of junctions. This study was performed with *n* = 6 for each condition.

### 4.9. Angiogenesis-Related Proteins

To compare the expression of angiogenesis-related proteins in HUVECs following ADSC-LPS-exo or ADSC-exo treatment, the Proteome Profiler Human Angiogenesis Antibody Array (cat. #AAH-ANG-2, RayBiotech, Dallas, TX, USA), which can detect 53 human angiogenesis-related proteins simultaneously with chemiluminescent detection reagents, was used to measure the protein expression in the cell lysates in quadruplicate, according to the manufacturer’s protocol.

### 4.10. Extraction of Exosomal Protein and iTRAQ Labeling

Exosomal proteins of ADSC-exo and ADSC-LPS-exo were purified using the T-PER tissue protein extraction reagent (78,510, Thermo Fisher Scientific, Waltham, MA, USA). Protein samples were desalted using Amicon^®^ Ultra-15 (Millipore) and quantified using the BCA protein assay (23,225, Thermo Fisher Scientific). For iTRAQ labeling, 25 µg of the protein samples were dried using SpeedVac and resuspended in the iTRAQ dissolution buffer, which included 0.5 M triethylammonium bicarbonate (TEAB; pH 8.5). Protein samples were reduced using the iTRAQ reduction buffer (tris-2-carboxyethyl phosphine, TCEP) at 60 °C for 30 min and then alkylated in the dark using iodoacetamide at 37 °C for the same amount of time. After protein digestion using sequencing-grade modified trypsin (V511A, Promega, Madison, WI, USA), the samples were dried using SpeedVac. Next, the peptides were reconstituted in the iTRAQ dissolution buffer and labeled using iTRAQ labeling reagents, according to the manufacturer’s instructions (Applied Biosystems Inc., Foster City, CA, USA).

### 4.11. Two-Dimensional Liquid Chromatography with Tandem Mass Spectrometry (2D LC-MS/MS)

The iTRAQ-labeled samples were analyzed using a Q Exactive^TM^ HF mass spectrometer (Thermo Fisher Scientific) coupled with an UltiMate™ 3000 RSLCnano HPLC System (Thermo Fisher Scientific). The iTRAQ-labeled peptides were pooled and desalted using Sep-Pak C18 cartridges (Waters, Milford, MA, USA). The desalted peptides were dried using SpeedVac and resuspended in 0.5% trifluoroacetic acid. The peptide mixtures were loaded onto an EASY-Spray™ C18 column (Thermo Fisher Scientific) and separated using a 0.1% formic acid solution with varying amounts of acetonitrile (5–80%). The top 15 abundant precursor ions within the 375–1400 *m*/*z* scan range were dynamically selected for further fragmentation in high collision dissociation (HCD) mode, with the normalized collision energy set to 33 ± 1%. In the full MS scan, the resolution was set to 60,000 at 200 *m*/*z*, AGC target to 3e^6^, and maximum injection time to 50 ms. For the MS/MS scan, the resolution was set to 15,000, AGC target to 5e^4^, and the maximum injection time was set to 100 ms. The release of the dynamic exclusion of selected precursor ions was set to 20 s.

### 4.12. Database Search and Protein Quantification

Raw MS data were examined using the Mascot search algorithm (version 2.5, Matrix Science) against the Swiss-Prot human protein database using Proteome Discoverer (version 2.1, Thermo Fisher Scientific) software. For protein identification, the search parameters were set as follows: carbamidomethylation at cysteine as the fixed modification, oxidation at methionine, acetylation at protein N-terminus, iTRAQ-labeled at peptide N-terminus, lysine residue as dynamic modifications, 10 ppm and 0.02 Da for MS/MS tolerance, and maximum missing cleavage sites with 2.

### 4.13. Construction of the Protein–Protein Interaction (PPI) Network and Identification of Hub Proteins

PPI network analysis [86,87] was used to distinguish critical hub proteins among groups of differentially expressed protein targets identified in the iTRAQ experiment. Therefore, the STRING database was used to conduct the PPI network analysis. PPI networks were constructed using Cytoscape 3.6.1, with nodes representing proteins and edges indicating simplifications of interactions between nodes in the network for graphical representation.

### 4.14. Statistical Analysis

All results are presented as the mean ± standard error. An overall analysis of the differences between group means was performed using a one-way analysis of variance (ANOVA), followed by a post hoc Fisher’s least significant difference test. Statistical significance was set at *p* < 0.05.

## 5. Conclusions

This study revealed that human ADSCs stimulated with LPS secrete exosomes that enhance angiogenesis in HUVECs. This phenomenon is related to the activation of CREB, AP-1, and NF-κB signal transduction pathways and IL-8 production in recipient HUVECs after ADSC-LPS-exo treatment. Pre-treatment of ADSCs with LPS can produce exosomes that carry potent molecules for enhanced angiogenesis. Proteomic analysis of ADSC-exo revealed the notable presence of HDAC, APP, and ITGB1, which may be potential candidates for exosome-mediated enhanced angiogenesis.

## Data Availability

Not applicable.

## Supplementary Materials

The following are available online at https://www.mdpi.com/article/10.3390/ijms22168877/s1.
